# Supplementary material for: Gut microbiota associations with common diseases and prescription medications in a population-based cohort
Source: Nat Commun. 2018 Jul 9;9:2655. doi: 10.1038/s41467-018-05184-7 (PMC6037668; doi:10.1038/s41467-018-05184-7)

Gut microbiota associations with common diseases and prescription medications in a population-based cohort

Jackson et al.

Supplementary Fig. 1. Age and BMI distributions for the cases of each disease within the subset of TwinsUK individuals considered in the study. Points and bars represent mean and 95% confidence intervals of the mean for age and BMI per disease, red lines denote mean BMI and age across all individuals in the study.

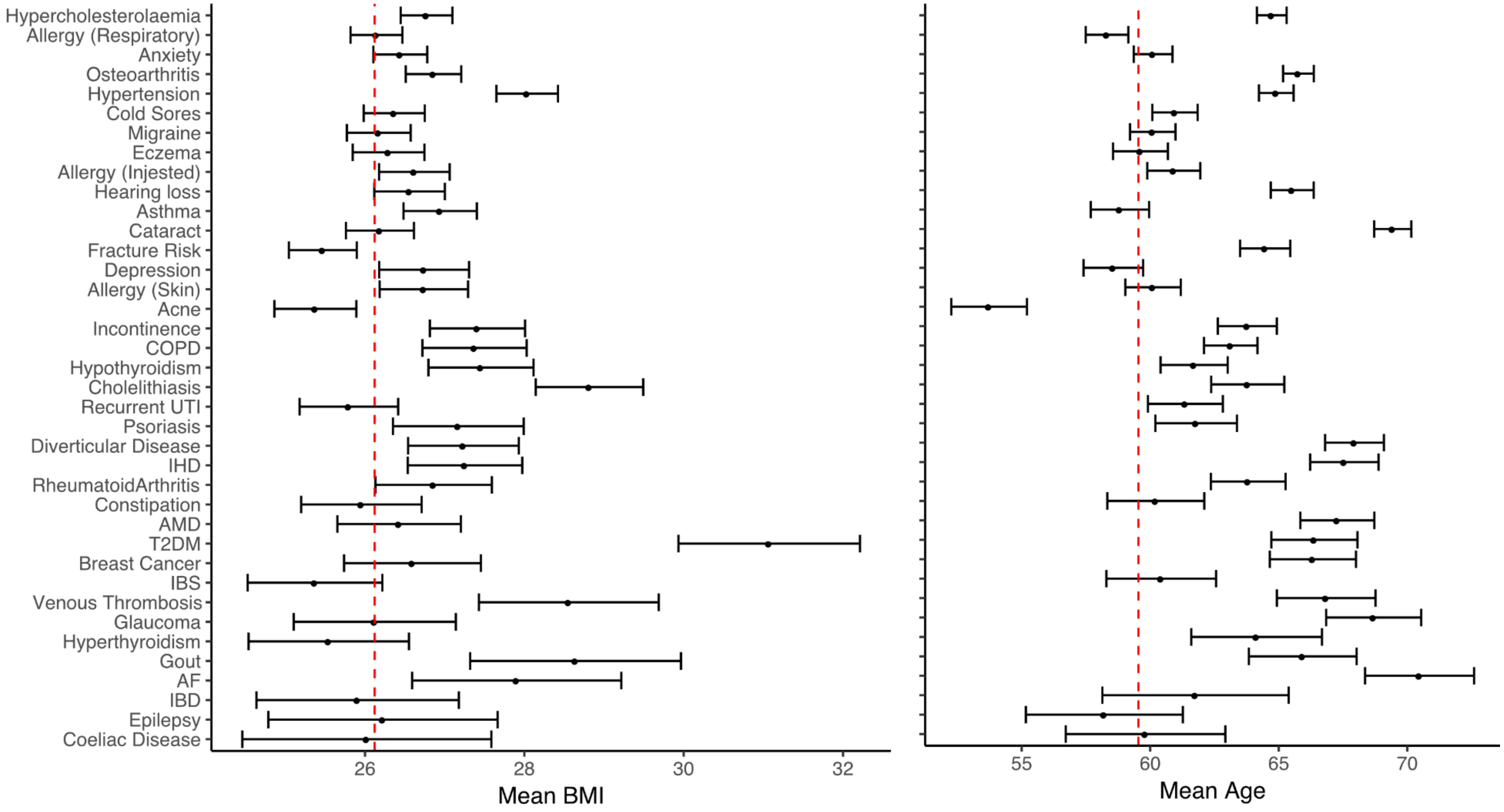

Supplementary Fig. 2. Disease correlation and gut microbiota associations plots as in Figure 1 but also including obesity and showing results from analyses without inclusion of BMI as a covariate.

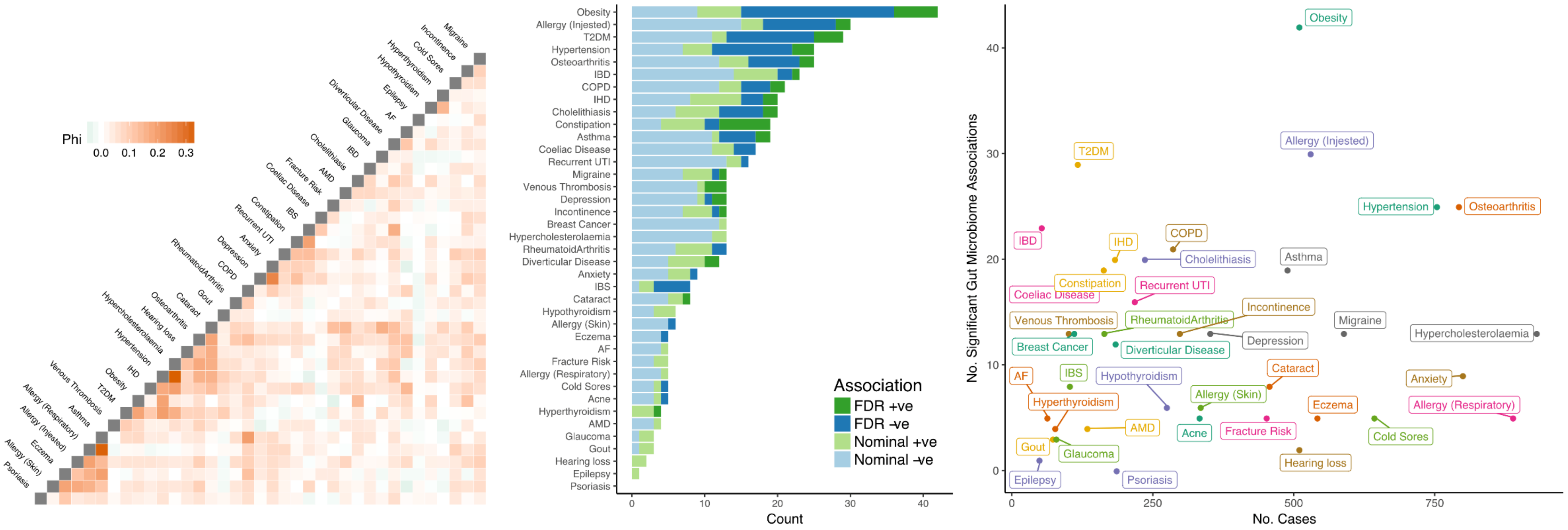

Supplementary Fig. 3. Plot showing the correlation of beta coefficients and p-values between the disease association results when modelling with different covariates. Labels represent which covariates were considered. Lower triangle shows a scatterplot of the values, the diagonal a density plot of the distribution of values, and the upper triangle shows Pearson correlation coefficients.

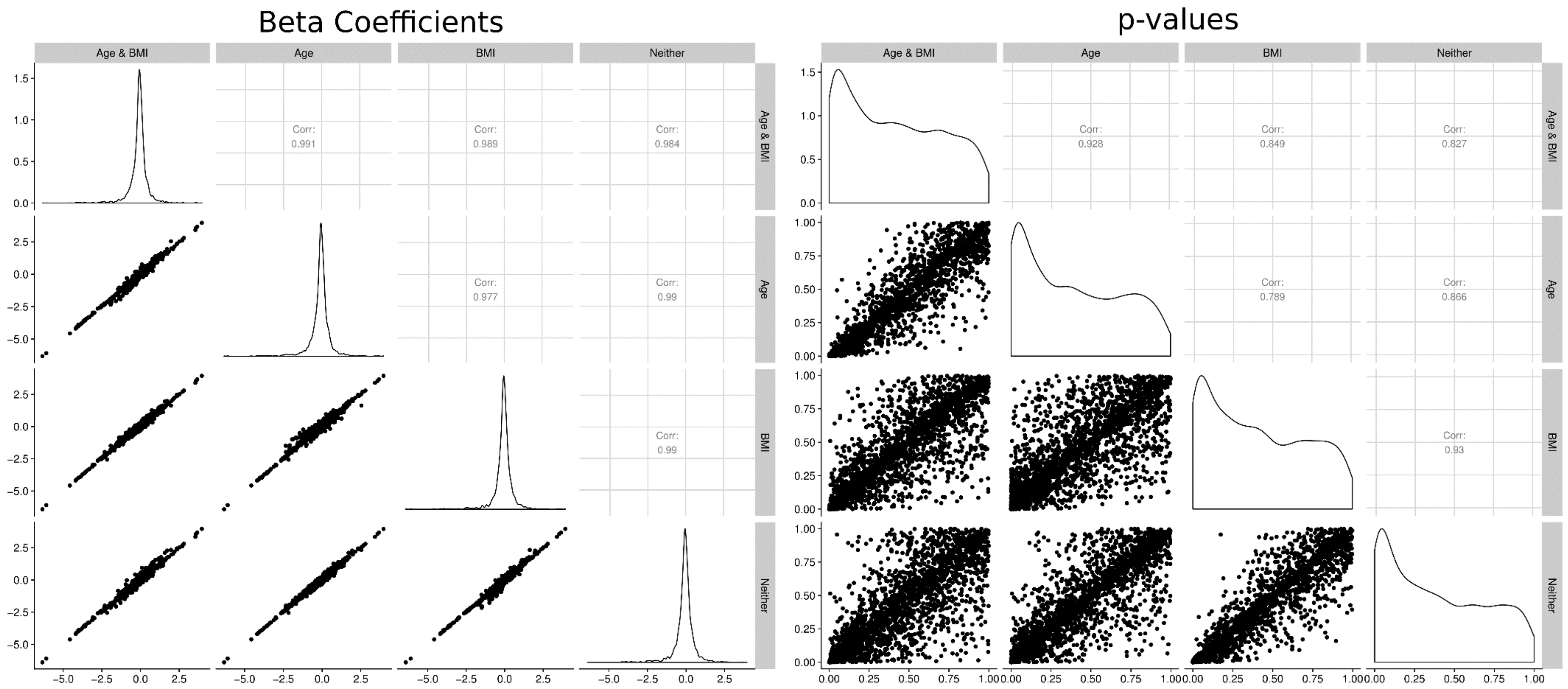

Supplementary Fig. 4. Heatmap of disease association results as in Figure 2 but considering the associations of non-specific genera as defined in the meta-analysis of Duvallet *et al*. 2017[2].

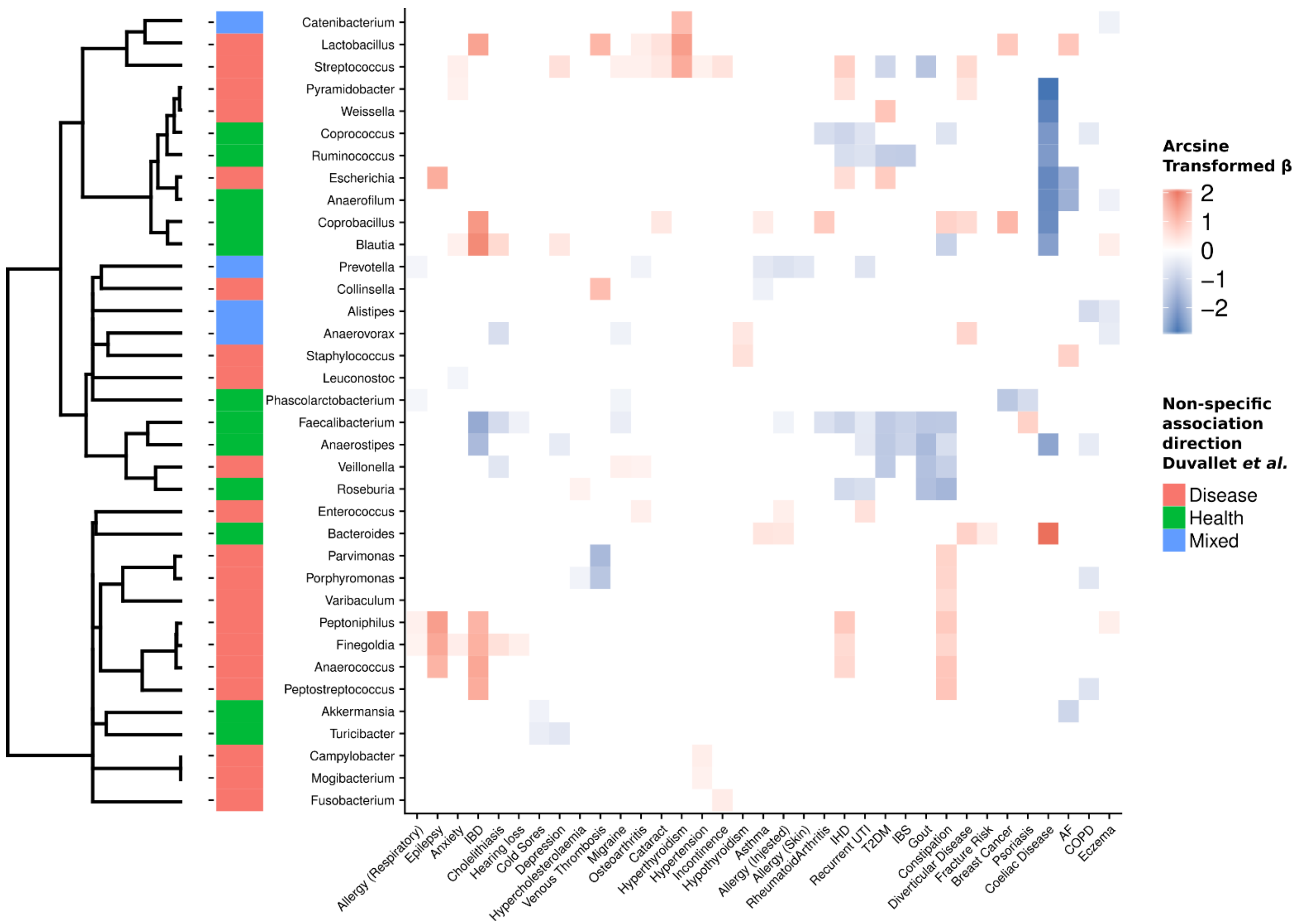

Supplementary Fig. 5. Gut microbiota associations across medications. Both medications and microbiota traits have been clustered based on cosine distances generated from the beta coefficients of all nominally significant (p<0.05) associations. Beta coefficients have been arcsine transformed for visualisation. Non-significant associations have been scored 0 and hence coloured white. Medications or microbiota traits with no significant associations are not shown.

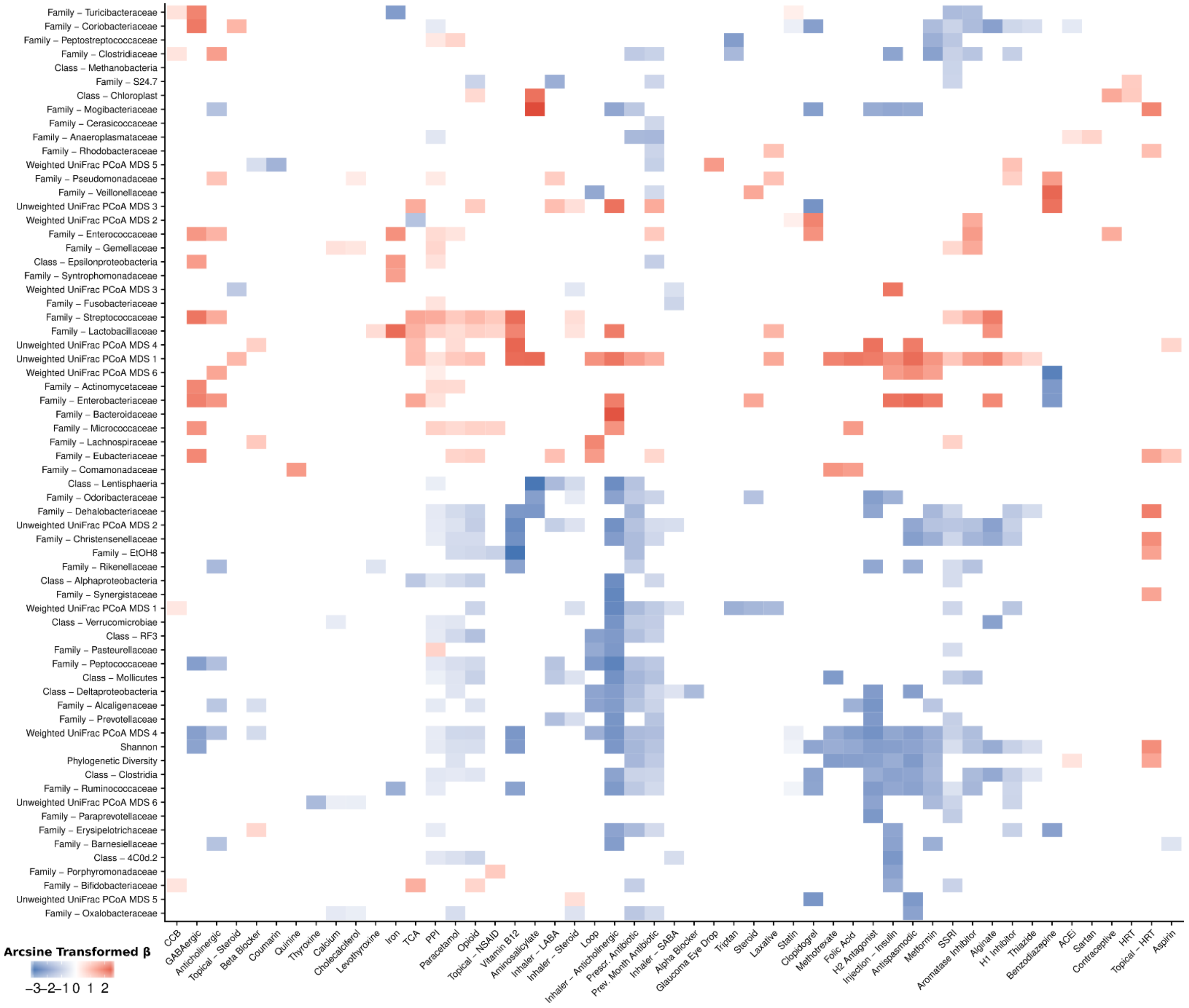

Supplementary Fig. 6. Plot of disease-medication pair correlations versus their overlap in gut microbiota associations as in Figure 4 but with all points annotated.

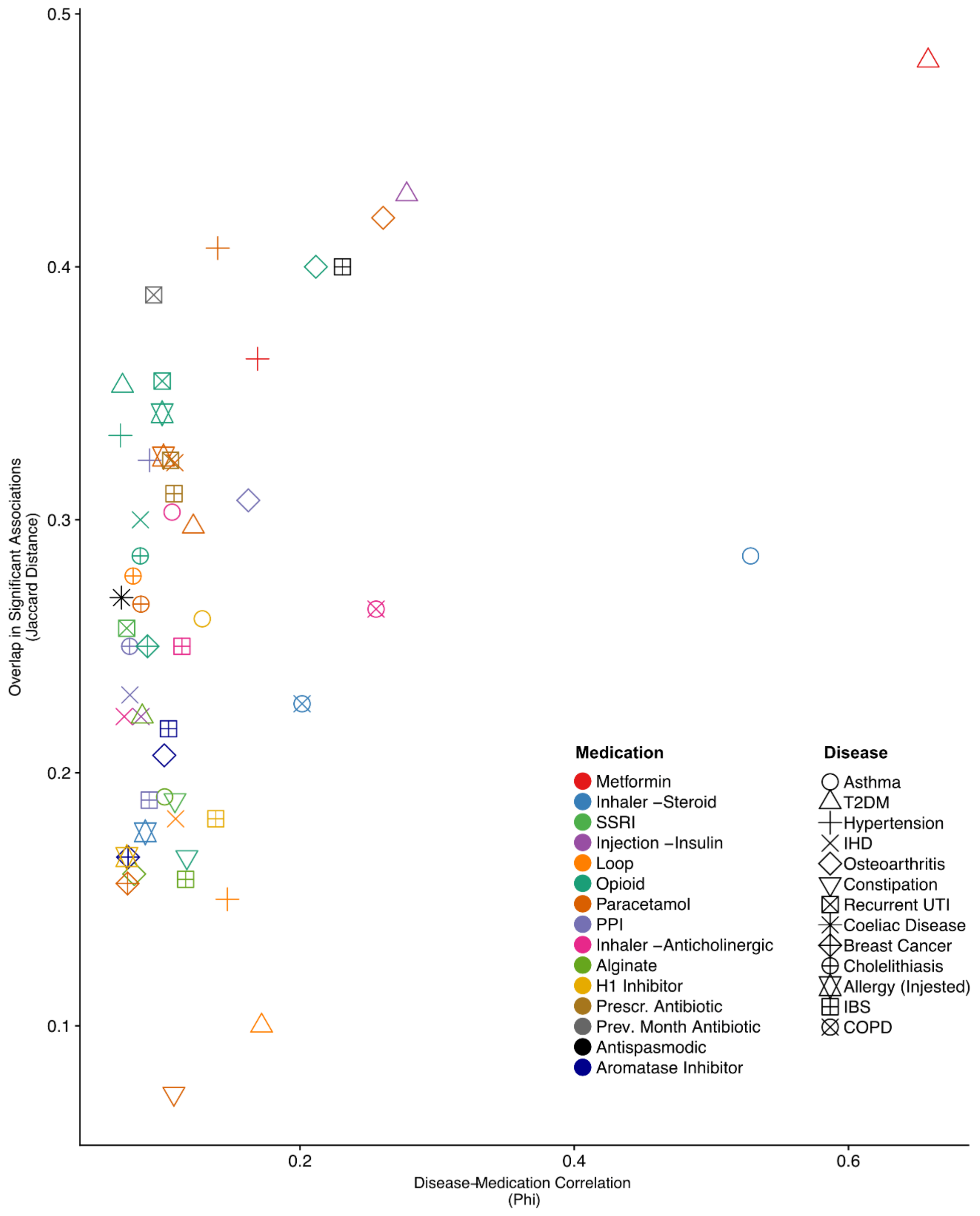

Supplementary Fig. 7. Bar plots showing the number of questionnaires used to score diseases and their time difference, in years, from faecal sampling, shown for each questionnaire considered. Most diseases were scored from questionnaires BCQ and Q11A.

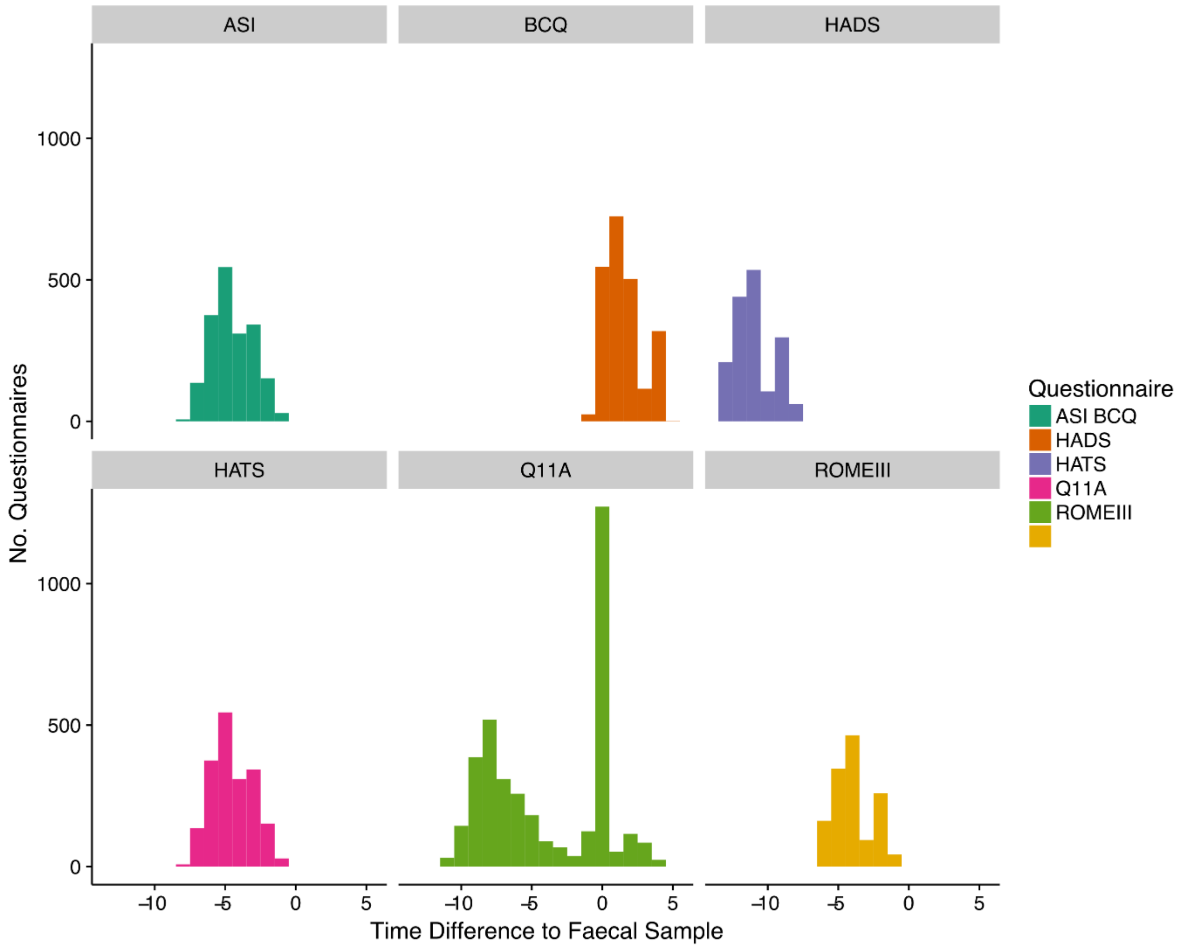

Supplementary Fig. 8. Scree plots showing the variance explained by axes in principle coordinates analyses of the weighted and unweighted UniFrac measures. The first six were considered in both cases as the variance explained plateaued at this point.

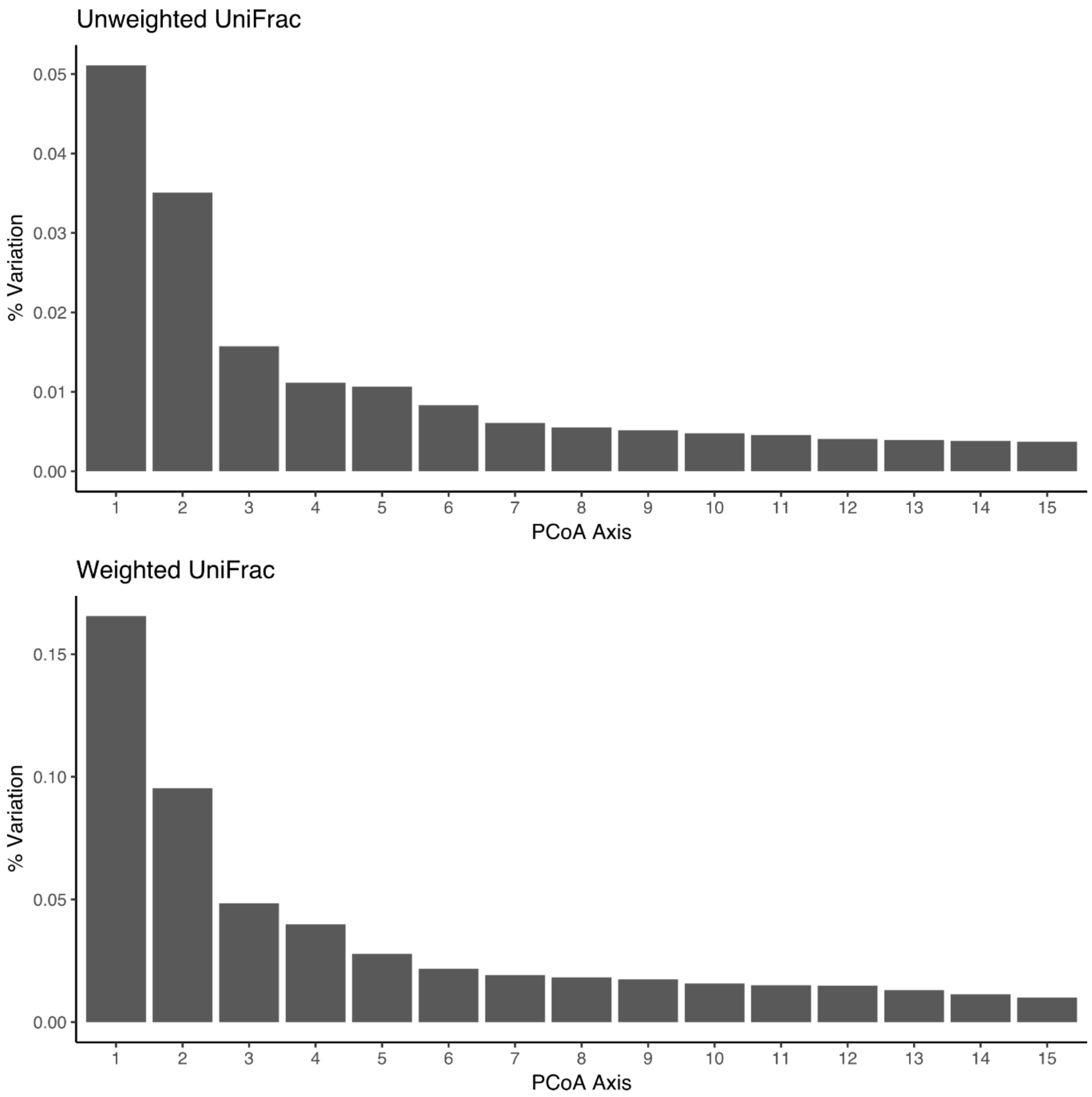

Supplement: Supplementary file 1 — Supplementary Information [file 41467_2018_5184_MOESM1_ESM.pdf]
