# Supplementary material for: Gut microbiota associations with common diseases and prescription medications in a population-based cohort
Source: Nat Commun. 2018 Jul 9;9:2655. doi: 10.1038/s41467-018-05184-7 (PMC6037668; doi:10.1038/s41467-018-05184-7)
Supplement: Supplementary file 3 — Description of Additional Supplementary Files [file 41467_2018_5184_MOESM3_ESM.pdf]

Description of Additional Supplementary Files

File Name: Supplementary Data 1
Description: Common diseases and prescription medications (found in at least 1% of cohort investigated) within the TwinsUK cohort.

File Name: Supplementary Data 2
Description: Mapping of the 206 gut microbiome features considered to their marker in the 68 marker features used in analyses. The marker is the one selected to have a Spearman correlation of at least 0.8 to the trait, although a trait could also be this correlated with multiple markers.

File Name: Supplementary Data 3
Description: Results from logistic regression analysis of diseases versus microbiome markers.

File Name: Supplementary Data 4
Description: Results from logistic regression analysis of disorders versus microbiome features only adjusting for BMI, age, or neither.

File Name: Supplementary Data 5
Description: Results from logistic regression analysis of prescription medication-classes versus microbiome features.

File Name: Supplementary Data 6
Description: Table showing the overlap of data used to calculate the correlations between the diseases and medication use in Figures 1b and 3b.
